# Association analysis of monoamine oxidase A gene and bipolar affective disorder in Han Chinese

**DOI:** 10.1186/1744-9081-4-21

**Published:** 2008-05-24

**Authors:** Yi-Mei J Lin, Fabian Davamani, Wei-Chih Yang, Te-Jen Lai, H Sunny Sun

**Affiliations:** 1 Institute of Molecular Medicine, National Cheng Kung University Medical College, Tainan, Taiwan; 2 Institute of Biomedical Sciences, Academia Sinica, Taipei, Taiwan; 3 Institute of Medicine, Chung Shan Medical University, Taichung, Taiwan; 4 Department of Psychiatry, Chung Shan Medical University Hospital, Taichung, Taiwan

## Abstract

**Background:**

Monoamine oxidase A (MAOA) is a mitochondrial enzyme involved in degrading several different biological amines, including serotonin. Although several pieces of evidence suggested that MAOA is important in the etiology of bipolar affective disorder (BPD), associations for markers of the MAOA gene with BPD were not conclusive and the association has not been investigated in Taiwanese population. This study was designed to illustrate the role of MAOA in the etiology of BPD in Han Chinese.

**Methods:**

Two markers, a dinucleotide polymorphism in exon 2 and a functional uVNTR on the promoter of the *MAOA *gene, were used to study the genetic association in 108 unrelated patients with BPD and 103 healthy controls. Allelic distributions of two polymorphisms were analyzed and, caused the MAOA located at X chromosome, haplotype association was performed using haplotype unambiguously assigned in male participants.

**Results:**

While no difference in allelic distributions of two MAOA polymorphisms was found, the risk haplotype 114S was associated with BPD in male patients (*P *= 0.03). The significance, however, was not found in female patients with 114S haplotype.

**Conclusion:**

Results from this study suggest that MAOA may have a gender-specific and small effect on the etiology of BPD in Taiwan. Due to the limited sample size, results from this study need to be confirmed in replicates.

## Background

Bipolar affective disorder (BPD) is a common severe mood disorder characterized by manic and depressive episodes; it has an estimated lifetime prevalence of 0.1~1% in various populations, including Chinese [1]. In spite of previous studies (review by [2]) strongly suggested that genetic factors were involved in the etiology of BPD, the classical strategy of linkage analysis has been fraught with difficulty in searching for the predisposing genes. As the "monoamine hypothesis" proposes that the underlying biological basis for depression is depletion of central noradrenergic, serotonergic, and dopaminergic systems [3], thus genes implicated in the metabolism or kinetics of monoamine neurotransmitter system, including the serotonin (5-HT) and dopamine (DA), are good candidates for studying the association with affective disorder [4].

Monoamine oxidase (MAO) is an enzyme expressed in the outer mitochondrial membrane; it catalyzes the degradation of biological amines [5]. There are two forms of monoamine oxidase, MAOA and MAOB, each with a different substrate and inhibitor specificities. The two MAO genes are localized tail to tail on Xp11.4-p11.23, identified using *in situ *hybridization [6], and span at least 60 kb. They consist of 15 exons and have identical exon-intron organization [7]. Several pieces of evidence have suggested that MAOA in particular is important in human behavior and physiology. The first association of the MAOA gene with psychiatric disease was reported in a study of a large family in Holland [8]; all the affected males showed characteristically abnormal behavior: aggression and sometimes violence. Using genetic linkage analysis, researchers assigned the locus for this disorder to an MAO gene mutation on the X chromosome. MAOB activity was normal in this family; the unusual behavior pattern was traced to a mutation in MAOA [8,9]. The *MAOA*-gene-knockout mice showed behavioral alteration [10]. MAOA inhibitors have been effectively used in clinical trials to treat BPD, and MAOA is a target for some antidepressant drugs [11,12]. These studies strongly suggested the possible involvement of MAOA in the etiology of BPD. Polymorphisms of the *MAOA *gene have been used to extensively study its association with several psychiatric conditions, such as BPD [13], depression [14], and aggression-related traits [15]. These polymorphisms includes a highly polymorphic dinucleotide (CA) microsatellite repeat in the second exon [16], a 23-bp VNTR in intron 1 [17], a 30-bp functional uVNTR (MAOA-uVNTR) that is ~1.2 Kb upstream of the coding region [18], an RFLP polymorphism (*Fnu4H1 *and *EcoRV*) [19], and some single nucleotide polymorphisms (SNPs) including rs1799835, rs6323, and rs1465108 [20,21].

The association of MAOA polymorphisms with BPD has been contradictory in different populations. For example, while studies conducted in Germany [22], the U.S.A. [23], Canada [24], and China [25] have shown no associations in their overall samples, positive associations have been reported from studies performed in Japan [26] and the U.K. [13]. Furthermore, studies done in France and Switzerland [27] and the U.K. [28] have reported positive associations in females but not in males. Another study in Japan revealed no association between MAOA and BPD for the overall sample as well as for either sex studied separately [29]. To illustrate the role of MAOA in the pathogenesis of BPD patients in Taiwan, we have conducted a genetic association study and analyzed the distributions of both promoter uVNTR and the (CA) repeat polymorphisms in BPD patients and controls. Further examination into the effect of risk haplotype has taken advantage of the fact that the *MAOA *gene is on X chromosome; therefore males are hemizygotes and the haplotype can be unambiguously assigned in male individuals. Our data revealed a risk haplotype association of MAOA polymorphisms and BPD in male patients only thus it suggests that MAOA may have a gender-specific and small effect in the etiology of BPD in Taiwan.

## Methods

### Sample collection and DNA preparation

The subjects were all Han Chinese who had been collected, beginning in 1998, for series studies investigating the involvement of serotonergic genes in the pathogenesis of BPD [30-33]. Patients and controls were interviewed by experienced psychiatrists after the study procedure had been fully explained and information on general demographic data, such as age, sex, and ethnicity, was obtained. In Briefly, 108 (59 males and 49 females) unrelated BPD patients were recruited from direct clinical interviews by a treating clinician according to the DSM-IV criteria for diagnoses of lifetime major depressive disorder and bipolar I. Additional information required to reach a diagnosis was also collected from all clinical and hospital records where available. Furthermore, information on family history of the disease and co-morbidity with other psychiatric disorders, neurological disorders, or medical problems were also collected. Only one patient was found to have co-morbid psychiatric condition with alcohol abuse. Although about 10% of the patients have medical problems like hypertension or diabetes, the diseases are common in general population as well. Thus the information on co-morbidity was not considered as inclusion or exclusion criteria in this study. One hundred three (55 males and 48 females) controls were recruited from volunteers with no family or personal history of major affective disorder and were matched to patients based on ethnic or geographic origin, sex, and age. The gender ratios are identical in both case and control groups (male to female is 53% to 47%). This study was approved by the University Ethics Committee and the standards established by the National Science Council, Taiwan. Written informed consent was obtained from all participants.

Genomic DNA was prepared from approximately 10 ml of peripheral blood using the standard salt-precipitation method [34] or a kit (InstaGene Whole Blood kit; Biorad, Hercules, CA, USA) according to the manufacturer's protocol.

### Polymorphism genotyping

The forward primer 5'-ACAGCCTGACCGTGGAGAAG-3' and reverse primer 5'-GAACGGACGCTCCATTCGGA-3' were used to amplify a fragment that contained the 30-bp repeat uVNTR polymorphisms [18]. PCR was performed in a 25-μl reaction containing 100 ng of genomic DNA, 250 μM of dNTPs, 0.2 μM of primers, and 0.5 units of Taq DNA polymerase (Promega, Madison, WI, USA) in 1× buffer with 1.5 mM of MgCl_2_. The condition set in the thermocycler was pre-denaturation for 3 min at 95°C, 1 min at 95°C, 1 min at 64°C, and 1 min at 72°C for 35 cycles, and then a final extension for 5 min at 72°C. The PCR products were separated using electrophoresis on a 2.5% agarose gel, visualized using ethidium bromide staining, and documented.

The MAOA-CA microsatellite polymorphism was amplified with the primer set FAM-5'-AGAGACTAGACAAGTTGCAC-3' and 5'-CACTATCTTGTTAGCTCAC T-3' [16]. Amplification was performed in 10-μl reaction containing 40–50 ng of genomic DNA, 200 μM of dNTP, 0.15 μM of primers, and 0.25 units of Taq DNA polymerase (Promega) in 1× buffer with 1.5 mM of MgCl_2_. Cycling parameters were pre-incubation for 3 min at 95°C, and then 35 cycles of 30 sec at 95°C, 45 sec at 56°C, 45 sec at 72°C, and a final incubation step of 7 min at 72°C. The PCR products were analyzed using a genetic analyzer (ABI310; Applied Biosystems). Capillary electrophoresis was performed by mixing 1 μl of the PCR product mix with 11.5 μl of formamide and 0.5 μl of size markers, heating the mixture at 95°C for 5 min, and then quickly cooling it on ice. Fourteen microliters of each sample was loaded onto a capillary polymer and run for 30 min. Products of different size were separated using polymer capillary electrophoresis, and the length of each PCR product was determined from the size markers. Data were collected and analyzed using computer software (ABI GeneScan; Applied Biosystems). To avoid genotyping errors, genotypes were scored independently by two investigators and checked for confirmation. In addition, discrepant genotypes were retyped to ensure the correct genotypes for final data analysis.

### Statistical analysis

Allelic distributions between patients and matched controls were analyzed using the χ^2 ^test and Fisher's exact test (SAS, version 8; SAS, Cary, NC, USA). Hardy-Weinberg Equilibrium was separately calculated for each gender and examined by χ^2 ^test implemented in the SAS program. For allelic distributions analysis, MAOA-CA and -uVNTR polymorphisms were grouped into several classes to minimize the allele number. Four major alleles of MAOA-CA with a frequency larger than 5% were chosen, and the other rare alleles (frequency less than 5%) were pooled together as a single allele. MAOA-uVNTR was classified in accordance with a previous taxonomy [18]. In addition, the linkage disequilibrium estimation (LD) and haplotypes construction using the expectation-maximization (EM) method were performed in female groups (SNPAlyze: SNP and Disease Association Analysis software version 4.1; Dynacom Co., Ltd. Kanagawa, Japan). Odds ratio and power estimation were estimated using SAS and Power Analysis and Sample Size (PASS) 2005 software (NCSS, Kaysville, UT, USA), respectively. The significance level for all statistical tests was 0.05 and we applied Bonferroni corrections for multiple tests in the association study.

## Results

### Polymorphism genotyping and single-locus association analysis

Two polymorphisms, MAOA-CA and -uVNTR, were genotyped in our case-control participants. For the microsatellite marker MAOA-CA, we found 9 alleles ranging from 106- to 126-bp with 2 bases increments (alleles 126–114 correspond to alleles A0–A6 of [28]). Interestingly, we found no participant with 122-bp and 124-bp alleles. For MAOA-uVNTR in the promoter region, we identified 3 alleles corresponding to 3, 4, and 5 repeats in our population. The distributions of alleles for each polymorphism are presented in Table 1. The genotype frequencies of MAOA-CA (9 alleles) and MAOA-uVNTR (3 alleles) for both genders in the patient and control groups were all in Hardy-Weinberg equilibrium (data not shown).

**Table 1:** Distribution of MAOA – CA and uVNTR alleles in 103 controls and 108 BPD patients.


	Patients (frequency)			Controls (frequency)		
		
Allele	M^a^	F^a^	Total	M^a^	F^a^	Total

CA(bp)^b^						
116	19 (0.42)	30 (0.37)	49	16 (0.41)	41 (0.47)	57
114	7 (0.15)	11 (0.14)	18	1 (0.03)	8 (0.09)	9
110	8 (0.17)	15 (0.19)	23	10 (0.25)	14 (0.16)	24
108	8 (0.17)	20 (0.25)	28	9 (0.23)	18 (0.20)	27
Others	4 (0.09)	4 (0.05)	8	3 (0.08)	7 (0.08)	10
Total	46	80	126	39	88	127
uVNTR^c^						
Short	32 (0.60)	49 (0.52)	81	25 (0.48)	49 (0.58)	74
Long	21 (0.40)	45 (0.48)	66	27 (0.52)	35 (0.42)	62
Total	53	94	147	52	84	136

^a^M, Males; F, Females.

^b^Distribution of CA dinucleotide polymorphisms. Common alleles (frequency more than 5%) and the combined rare alleles were shown.

^c^Distribution of uVNTR polymorphism. The short allele is the 3 repeats, and the long allele includes 4 and 5 repeats.

Allele frequencies of MAOA-CA and -uVNTR polymorphism were compared between the BPD patients and matched controls using the χ^2 ^test and Fisher's exact test. Rare alleles of MAOA-CA (frequency less than 5%) were pooled together as a single allele, and MAOA- uVNTR was classified in accordance with a previous classification [18] that repeats 4 and 5 were combined to be the long allele. No differences were observed in single alleles or global distributions of MAOA-CA and -uVNTR. The allelic distributions of each marker are listed in Table 1.

### Linkage disequilibrium measurement and risk haplotype analysis

Using haplotype analysis to investigate genetic association has some advantages. First, it allows multiple potentially causal loci to be tested simultaneously for association. Second, haplotypes may be proxies for untyped causal markers [35]. Once the haplotypes are defined, it is straightforward to examine a subset of SNPs and disease association. We select the 114 allele of MAOA-CA and the short allele of functional MAOA-uVNTR to comprise a 2-allele haplotype to examine its risk for BPD. The 114S haplotype determined by combination of the most risk allele of MAOA-CA (114 allele) and the transcriptionally less active short allele of MAOA-uVNTR. Since *MAOA *gene locates on X chromosome, haplotypes were assigned to each individual male participant explicitly. The distribution of the 114S haplotype was significantly different between male patients and controls (Table 2; *P *= 0.01; Power = 75% when α = 0.05) and associated with a risk to BPD (OR = 12.64, 95% CI = 0.69–232.88). To test whether the 114S haplotype has distributed differently in female groups, the linkage disequilibrium coefficient values (D') of two polymorphisms were calculated for females in patients (D' = 0.88) and controls (D' = 0.78). The 114S haplotype was estimated to present 8 and 6 times in patients and controls, respectively, and no differences in haplotype distribution between two groups was evident in female (Table 2; *P *= 0.72). After the correction for twice testing that were conducted in each gender, the difference in male group is still significant (*P *= 0.03).

**Table 2:** Risk haplotype distribution in BPD patients and controls.


	Haplotype 114S^a^	Patients (freq.)	Controls (freq.)	*P *value	Odds Ratio (95% Confidence Interval)

Males		6 (0.14)	0 (0.00)	0.01	12.64 (0.69–232.88) ^b^
Female		8 (0.08)	6 (0.06)	0.72	1.22 (0.41–3.67)

^a^Power for the 114S haplotype distribution is about 74.9% with α = 0.05.

^b^Since odds ratio is undefined with zero cell, adding 0.5 to each cell was used for calculate the odds ratio for 114S haplotype.

## Discussion

BPD is a common disease that is genetically heterogeneous with the same clinical manifestation caused by different susceptibility genes or a combination of low penetrating genes with possible environmental effect. Analysis using mathematical modelling suggests the heterogeneous basis for the inheritance of bipolar disorders [36]. For investigating these complex phenotypic traits like BPD, current studies suggest that using association analysis can have acceptable power to identify these small genetic effects [37].

This study aims to investigate the MAOA gene effect on BPD etiology in Han Chinese. The genetic association between two polymorphisms of the MAOA gene and BPD was conducted in 108 BPI patients and 103 normal controls. To overcome the sample size limitation for detecting small risk effect, we consider the two risk allele at the same time and examine if there are any additive or interactive risk for BPD. Growing evidences have supported the existence of higher-order risk interactions between or within genes to cause common and complex diseases [33,38,39]. Previous study also showed a combination of several common variants, that an association with disease at single-locus levels are not significant, has a dramatic impact on the etiology of complex diseases [33]. Using the haplotype based interaction-considered approach; we successfully identified a risk haplotype for BPD.

Significant risk haplotype effect was detected in male groups but not in female groups. Our hypothesis of the male-specific effect is the male only have single copy of *MAOA*, thus the carrying of risk haplotype lead more contribution to the diseased state. The gender effect of MAOA in various psychiatric diseases has been discussed for a long while. The first mutation in MAOA related to abnormal behavior was seen in several males [8]. The MAOA deficiency was associated with increased aggression; a similar finding was made in male mice [10]. In addition, many studies have reported finding significant gender-specific associations with psychiatric diseases [14,27,40] including BPD [27,28]. Collectively, these results suggest that the MAOA mechanism underlying the etiology of BPD might be different in males and females.

Just like other common psychiatric syndromes, BPD is known to have complex etiology including effect of multiple pathogenic genes and non-genetic factors [41]. The controversial results from previous association studies in various populations [22,25,26] suggest the MAOA gene and/or its interacting factors may have ethnic specificity in BPD etiology. Although the risk haplotype of *MAOA *only carried by few BPD patients and suggests that MAOA gene may have only a small effect on the pathogenesis of BPD, other studies have correlated MAOA with many human behavioral traits that belong to the same spectrum with BPD, including aggression [8], anxiety [42], depression [14], and suicide [43]. Furthermore, the MAOA-knockout mice showed aggressive behavior, which indicates a major effect of MAOA deficiency. Taken together, these results strongly suggest that MAOA is indeed involved in the spectrum of diseases related to abnormalities of the central nervous systems; however, the effect of MAOA on different diseases seems to be dissimilar.

Earlier studies indicated that there were 5 alleles of the uVNTR polymorphism (2, 3, 3.5, 4, and 5; 30-bp repeats), allele 3, 3.5, 4, and 5 were found in German population [44], and in White/Non-Hispanic and African-American/Black genotyping studies [18]. Another rare allele that has two 30-bp repeats was found in Japanese, British Caucasian, Swedish and Italian population [44-47]. Furthermore, the long alleles (4 and 5, 30-bp repeat) have been reported to be transcriptionally more active than the short allele (3, 30-bp repeat) [18]. In the present study, we detected 3 alleles (3, 4, and 5) in people of Han Chinese origin in Taiwan, and the major alleles are 3 and 4 repeats. The observed allele distribution in this study is similar to another report from Taiwan [14] and to a report from the People's Republic of China [48]; all three together suggest that this unique distribution pattern may be specific to ethnic Chinese.

Strong linkage disequilibrium was observed between the MAOA-uVNTR and MAOA-CA markers because these two markers are only 24 Kb apart. The linkage disequilibrium D' value suggests only a negligible level of recombination in the population studied. The shorter MAOA-CA alleles were found most frequently on the chromosome carrying MAOA-uVNTR allele 4 or 5, whereas the longer MAOA-CA alleles appeared most frequently on the chromosome carrying MAOA-uVNTR allele 3 (data not shown). This result, however, is different from the previous report, in which strong linkage disequilibrium was observed for long MAOA-uVNTR with long MAOA-CA alleles and short MAOA-uVNTR with short MAOA-CA alleles in the U.S. population [18]. The discrepancies in allelic distribution and phase of linkage disequilibrium might be attributable to ethnic differences.

In the past few years, our series studies focus on the association of serotonergic genes with BPD, including the serotonin synthetic enzymes TPH1 [31] and TPH2 [33], the serotonin transporter SLC6A4 [30], and several serotonin receptors (HTRs) [32]. Those results together with our findings in the current study support our hypothesis that the serotonergic system has a major impact on the pathogenesis of BPD. Although each of genes involved in the serotonergic system may contribute only a small to modest effect, their co-contribution with the environment or other genetic or epigenetic factors may result in BPD. Although our study suggested that MAOA might have a small effect on susceptibility to BPD, the chance of false positives cannot be excluded because of the sampling limitations (i.e., small sample size and subject assessment lacked support by structured interviews). Because of the limitations, further investigations with larger populations are required to elucidate the significance of MAOA on the pathogenesis of BPD in Taiwan.

## Conclusion

Results from this study suggest that MAOA may have a gender-specific and small effect on the etiology of BPD in Taiwan. We found that all male individuals carried 114S risk haplotype appear in BPD group, but equally distribute on female patient and control groups. But due to the limited sample size, results from this study need to be confirmed by further investigations with larger populations.

## Competing interests

The authors declare that they have no competing interests.

## Authors' contributions

YMJL analyzed the data and drafted the manualscript, FD and WCY performed the genotyping, TJL recruited the subjects, and the corresponding author HSS designed the study, conducted experiments and finalized the manualscript. All authors have read and given final approval of the final manuscript.
